# Protective effects of *Angelica dahurica* polysaccharide on oxidative stress and liver injury induced by ovariectomized/D-galactose in rats

**DOI:** 10.1080/13510002.2026.2674473

**Published:** 2026-05-20

**Authors:** Xue-qin He, Yi-zhang Li, Li-ba Xu, Chun-feng Wu, Du-dong Wei, Hao-yu Liu, Fen Qiu

**Affiliations:** a Guangxi University of Chinese Medicine, Nanning, China; b Key Laboratory of TCM Neuro-metabolism and Immunopharmacology of Guangxi Education Department, Guangxi University of Chinese Medicine, Nanning, China; c Guangxi Key Laboratory of Zhuang and Yao Ethnic Medicine, Guangxi University of Chinese Medicine, Nanning, China

**Keywords:** *Angelica dahurica*, polysaccharide, ovariectomized, D-galactose, oxidative stress, liver injury, Nrf2, HO-1

## Abstract

**Background:**

Oxidative stress-induced liver injury can progress to severe hepatic conditions. Polysaccharides are promising therapeutic agents, but the protective effects of *Angelica dahurica* polysaccharide (ADP) against this injury remain unclear.

**Purpose:**

This study was conducted to elucidate ADP's protective effects and underlying molecular mechanisms in Ovariectomize/D-galactose (OVX/D-Gal) rats.

**Methods:**

Fifty female OVX rats and ten sham-operated (SO) rats were randomly divided into six groups. Except SO group, rats were administered the corresponding doses of ADP and resveratrol respectively, and the model was established by 49-day daily subcutaneous D-Gal injection. Serum and liver samples were collected to examine the hepatic histopathological changes, oxidative stress-related biomarkers, inflammatory factors, and protein and gene expression levels.

**Results:**

ADP reduced serum ALT and AST levels (*P* < 0.01) and improved liver histology. ADP increased SOD and GSH-Px activities , decreased MDA levels (*P* < 0.05). ADP also lowered pro-inflammatory cytokines levels, upregulated Nrf2, HO-1, and NQO-1 expression, promoted Bcl-2 and suppressed Bax and cleaved caspase-3 (*P* < 0.05).

**Conclusions:**

ADP alleviated OVX/D-Gal-induced oxidative stress and liver injury by increasing antioxidant capacity, inhibiting apoptosis, attenuating inflammation, and activating Nrf2/HO-1/NQO-1 signaling pathway.

## Graphical abstract

**Figure:**
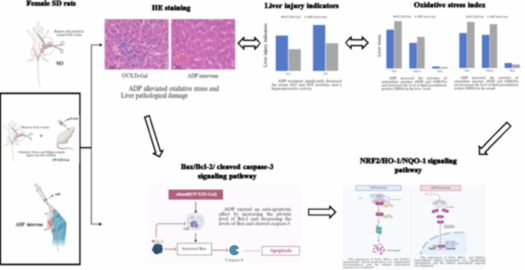

## 1. Introduction

The liver is the largest substantive organ in the human body; it plays a vital role in maintaining homeostasis through numerous complex physiological functions, including metabolism, detoxification, synthesis and secretion, immune defense, and storage [1,2]. However, the liver is highly susceptible to damage from factors such as alcohol, viruses, bacteria, and drugs [3]. If left uncontrolled and untreated, liver injury can progress to a variety of hepatic diseases, including fibrosis, dysfunction, cirrhosis, and liver failure, thereby compromising patient survival and quality of life [4]. At present, liver injury and related diseases pose a significant public health challenge worldwide. Approximately 2 million people die from liver diseases annually, accounting for 4% of the global mortality rate [5]. Oxidative stress is a key mechanism underlying liver injury, driven by an imbalance between oxidants and antioxidants [6,7]. When there is an excessive generation of oxidative free radicals or insufficient antioxidant capacity, the equilibrium shifts toward oxidants; these excess free radicals directly induce liver damage by promoting lipid peroxidation, inflammatory responses, and cell death. Persistent liver injury is a common pathway in the pathogenesis and progression of various liver diseases [8,9]. Thus, inhibiting oxidative stress and alleviating liver injury are important strategies for the prevention and treatment of liver diseases.

Estrogen plays a critical role in maintaining hepatic redox homeostasis. Studies have demonstrated that estrogen can upregulate the expression of antioxidant enzymes by activating estrogen receptor-mediated signaling pathways, thereby enhancing the liver’s resistance to oxidative stress [10,11]. Hepatic antioxidant defenses are significantly reduced under estrogen deficiency, as evidenced by decreased activity of antioxidant enzymes (e.g. superoxide dismutase [SOD] and glutathione peroxidase [GSH-Px]), and increased lipid peroxidation product accumulation. These effects markedly increase the liver’s susceptibility to various oxidative insults [12,13]. Thus, estrogen deprivation significantly exacerbates oxidative liver injury. D‑galactose (D-Gal) is a reducing monosaccharide sugar found in the body and in several foods. In mammals, D-Gal is typically metabolized and eliminated within 8 h of ingestion [14]. However, excessive administration of D-Gal can induce the production of reactive oxygen species (ROS) through various mechanisms, leading to oxidative stress and excessive proteins, lipids, and DNA peroxidation [15]. The liver, as the largest metabolic and detoxifying organ, is also a primary site of D-Gal-induced oxidative stress-related damage. Severe oxidative stress can damage hepatic protein and DNA structures, induce massive hepatocyte apoptosis, and impair the structure and function of liver tissue [16–18]. The ovary is the primary organ responsible for estrogen secretion. In rats, ovariectomy (OVX) and D‑Gal administration produce synergistic damaging effects by inducing estrogen deficiency and oxidative stress, mimicking the pathological characteristics of aggravated oxidative stress under estrogen decline. Thus, an OVX/D-Gal rat model is ideal for studying liver injuries [19,20].

Emerging evidence indicates that natural compounds confer hepatoprotective effects by modulating key signaling pathways. For example, graveoline alleviates acute liver injury by interfering with the JAK1/STAT3 signaling pathway [21], whereas total saponins from *Acanthopanax senticosus* effectively mitigate carbon tetrachloride (CCl₄)-induced acute liver injury in rats by modulating the PI3K/Akt and SIRT6/NF-κB pathways [22]. Similarly, the ethanolic leaf extract of *Lagerstroemia speciosa* attenuates acetaminophen-induced liver injury by coordinating the regulation of the NF-κB, Nrf2/HO-1, and Bax/Bcl-2 pathways [23]. In recent years, plant-derived polysaccharides have attracted increasing attention owing to their potent antioxidant and cytoprotective properties. Maca polysaccharides have been shown to suppress oxidative stress-induced neurotoxicity both *in vitro* and *in vivo* by enhancing antioxidant enzyme activities, scavenging ROS, and downregulating the p53/caspase-3 apoptotic cascade [24]. Furthermore, CHP‑N‑1, a neutral polysaccharide isolated from *Cimicifuga heracleifolia,* exerts hepatoprotective effects against CCl₄-induced liver injury by inhibiting the TLR4/NF-κB signaling pathway [25]. The protective role of polysaccharides against oxidative injury has been further substantiated in the D‑Gal-induced oxidative damage model, in which *Polygonatum sibiricum* [26], *Acanthopanax senticosus* [27], and saffron [28] polysaccharides have been shown to mitigate oxidative damage by enhancing antioxidant enzyme activity and alleviating oxidative stress.

*Angelica dahurica* polysaccharide (ADP) is a water-soluble polysaccharide isolated from the traditional herb and food source *Angelica dahurica* Radix; they exhibit diverse biological activities, including anti-inflammatory [29,30], antitumor [31], hepatoprotective [32], and antioxidant [33] effects. ADP comprises multiple monosaccharides, including rhamnose, arabinose, xylose, mannose, glucose, and galactose [34]. Previous studies have shown that ADP can ameliorate oxidative stress in the liver of type 2 diabetes mellitus rats and partially alleviate liver lesions [32]. However, the efficacy of ADP against oxidative stress and liver injury induced by OVX/D-Gal, as well as their underlying mechanisms, remains unclear. Specifically, it remains unknown whether ADP can alleviate this injury by regulating oxidative stress and inhibiting hepatocyte apoptosis. Thus, in this study, we established an OVX/D-Gal-induced liver injury rat model and assessed liver dysfunction by measuring the serum alanine aminotransferase (ALT) and aspartate aminotransferase (AST) levels. Systemic inflammation was assessed by measuring serum tumor necrosis factor-alpha (TNF-*α*), interleukin-1 beta (IL-1β), and interleukin-6 (IL-6) levels by enzyme-linked immunosorbent assay (ELISA). The antioxidant capacity of ADP was assessed by measuring SOD, malondialdehyde (MDA), and GSH-Px levels in serum and liver tissue. The activation status of the Nrf2/HO-1/NQO-1 signaling pathway was verified by measuring the messenger RNA (mRNA) and protein expression levels of nuclear factor erythroid 2-related factor 2 (Nrf2), heme oxygenase-1 (HO-1), and NAD(*P*)H quinone dehydrogenase 1 (NQO-1) in the liver tissue. Meanwhile, hepatocyte apoptosis was evaluated by examining the expression levels of apoptosis-related proteins, including Bcl-2-associated X protein (Bax), cleaved caspase-3, and B-cell lymphoma-2 (Bcl-2).

This study was conducted to investigate the protective effects of ADP against liver injury in OVX/D-Gal model rats and determine whether ADP exerts hepatoprotective effects by enhancing antioxidant capacity, reducing inflammatory responses, inhibiting apoptosis, and activating the Nrf2/HO-1/NQO-1 signaling pathway, thereby providing a theoretical basis for the clinical application of ADP.

## 2. Materials and methods

### 2.1. Reagents

ADP with 90.12% purity (Standard of Pharmacopeias, UV) was purchased from Xi’an Jiatian BioTechnology Co., Ltd. (Shanxi, China, JT20240820). D-Gal and Resveratrol were purchased from Shanghai Aladdin Biochemical Technology Co., Ltd. (Shanghai, China, J2430358, B2215709). Benzylpenicillin was purchased from Hebei Yuanzheng Pharmaceutical Co., Ltd. (Hebei, China, E8E230708). Kits for measuring ALT, AST, SOD, MDA, and GSH-px levels were purchased from Nanjing Jiancheng Institute of Biotechnology Co., Ltd., as was Coomassie blue (Jiangsu, China; 20250418, 20250419, 20250226, 20250417, 20250415, 20250417). The ELISA kits for TNF-*α*, IL-1β, and IL-6 were purchased from Biyabscience Biotechnology Co., Ltd. (Jiangsu, China; 260319008D1063, 260319008D0206, 260319008D0219). Anti-Bcl-2 and anti-NQO-1 antibodies were purchased from Proteintech Group, Inc. (Chicago, U.S.A.; 00156114, 10016784), and goat anti-rabbit IgG (H + L) HRP, HRP-conjugated goat anti-mouse IgG (H + L), anti-GAPDH, and anti-Bax were purchased from ABclonal Biotechnology Co., Ltd. (Hubei, China; 56j9958, 9300003001, 3600003301, 3600001937). Anti-cleaved-caspase3, anti-Nrf2, and anti-Keap1 were purchased from Affinity Biologicals (Canada, 4a60055, 31r1518, 85d1712), and anti-HO-1 was purchased from Abcam (UK, GR325245-19). The Total RNA Isolation Kit and Bicinchoninic Acid (BCA) Protein Assay Kit were purchased from BioSharp Biotechnology Co., Ltd. (Anhui, China, 3332424663x, 24098351). The PrimeScript RT reagent kit was purchased from Takara Biomedical Technology (Beijing) Co., Ltd. (Beijing, China, A011112A). TB Green TM Premix Ex TaqTM Ⅱ (Tli RNaseH Plus) was purchased from Takara Biomedical Technology (Beijing) Co., Ltd. (Beijing, China; AOF0472A). All other reagents and solvents were of analytical grade and used directly.

### 2.2. Animals and ethical approval

Female Sprague Dawley rats, weighing 171.2 ± 6.9 g, were obtained from the Hunan SJA Laboratory Animal Co., Ltd. (Hunan, China, Certificate No. SCXK [Xiang] 2021-0002). All animals were raised in a specific pathogen-free animal room at Guangxi University of Chinese Medicine. The rats were housed in standard IVC cages with a 12-h light–dark cycle. The room was temperature-controlled (20.0–25.0 °C) with a relative humidity of 40.0%–70.0%. The rats were given adequate access to food and water. All efforts were made to minimize suffering. All experiments were approved by the Animal Care and Use Ethics Committee at Guangxi University of Chinese Medicine (Approval No.: DW20241130-01) and conducted per the Guide for the Care and Use of Laboratory Animals.

### 2.3. Animal model

After a 1-week acclimation period, the experimental rats underwent bilateral OVX, and the sham-operated (SO) group (*n* = 10) underwent surgery without bilateral OVX. One week after surgery, the rats were randomly divided into six groups (10 rats per group). The first group (SO) received pure water (i.g.) for 7 weeks. The rats in the second group (OVX/D-Gal) were subcutaneously injected in the back with D-Gal (200 mg/kg/day) dissolved in 0.9% saline solution for 7 weeks [35]. The rats in the third group (OVX/D-Gal + Resveratrol) were subcutaneously injected with D-Gal as described above and received resveratrol (100  mg/kg) by gavage for 7 weeks [36]. The rats in the fourth group (OVX/D-Gal + ADP100) received D-Gal as described above, plus ADP (100 mg/kg) by gavage for 7 weeks. The rats in the fifth and sixth groups (OVX/D-Gal + ADP200 and OVX/D-Gal + ADP400, respectively) received the same treatment as those in the fourth group, except that the dose of ADP was increased to 200 and 400  mg/kg, respectively. On day 49, the rats were sacrificed under isoflurane anesthesia for experimental evaluation. Serum and liver tissue samples were collected for analysis, including pathological staining, quantitative real-time polymerase chain reaction (qRT-PCR), and Western blotting (WB).

### 2.4. Assessment of hepatic marker enzymes and inflammatory factors

Rat blood was collected before the animals were sacrificed and allowed to clot at 20.0–25.0 °C for 2 h. Serum was obtained by centrifugation at 3000 r/min for 10 min at 4 ℃ and stored at −20 °C until testing. Serum ALT and AST levels were measured using the respective kits according to the manufacturer’s instructions. Serum TNF-*α*, IL-1β, and IL-6 levels were measured using the ELISA kits according to the manufacturer’s instructions.

### 2.5. Assessment of oxidative stress

Rat liver tissue samples were collected after the animals were sacrificed. The tissue was then cut and accurately weighed. Saline was added at a ratio of weight (g) to volume (mL) of 1:9. A homogenate was prepared in an ice-water bath and centrifuged at 3000 r/min for 10 min. The supernatant (10% homogenate supernatant) was collected and stored at −20 °C for testing. SOD, MDA, and GSH-Px levels were measured in the serum and supernatants from homogenized liver tissue samples using a biochemical test kit according to the manufacturer’s instructions.

### 2.6. Histopathological evaluation

Liver samples were fixed in 4% paraformaldehyde. Sections (4 μm thick) from liver and cerebrum samples were stained with hematoxylin- eosin (HE), and analyzed under a light microscope (CX31, Olympus, Tokyo, Japan).

### 2.7. qRT-PCR

Rat liver tissue samples were stored at −80 °C, and total RNA was extracted using the Total RNA Isolation Kit per the manufacturer’s directions. The RNA was then reverse transcribed into cDNA using the PrimeScript RT reagent Kit. The mRNA expression levels of Nrf2, NQO-1, and HO-1 were determined by qRT-PCR. The relative expression levels of Nrf2, NQO-1, and HO-1 were calculated by determining the ratio of Nrf2, NQO-1, and HO-1 to *β*-actin (Table 1). All primers were designed and synthesized by Shanghai Shenggong Bioengineering Technology Service Co., Ltd., and were subsequently purified with ULTRAPAGE. The sequences are provided in Table 1.

**Table 1.** Sequences for real-time PCR.

	Forward primer 5'–3'	Reverse primer 5'–3'
*β*-actin	GGGAAATCGTGCGTGACATT	GCGGCAGTGGCCATCTC
HO-1	AGAGACGCCCCGAGGAAAATC	TGCCACGGTCGCCAACAG
NQO-1	GGCTGCTGTGGAGGCTCTG	TCCCCTGTGATGTCGTTTCTGG
NRF2	TGCCTTCCTCTGCTGCCATTAG	CCGTGCCTTCAGTGTGCTTC

### 2.8. Western blotting

The rat liver tissue samples stored at −80 °C were thawed, homogenized, and centrifuged. The protein concentrations in the supernatants were determined using a BCA assay. The protein samples were then electrophoresed via sodium dodecyl sulfate–polyacrylamide gel electrophoresis (SDS–PAGE) and transferred onto polyvinylidene fluoride membranes. The membranes were blocked with 5% (w/v) skim milk in Tris-buffered saline containing 0.1% Tween-20 (TBST) for 2 h at room temperature. After blocking, the membranes were incubated overnight at 4 °C with the following primary antibodies diluted in the TBST: GAPDH (1:200000), Bax (1:2000), Bcl-2 (1:2000), Cleaved-Caspase3 (1:2000), Nrf2 (1:2000), NQO-1 (1:5000), HO-1 (1:2000), and Keap1 (1:2000). Subsequently, the membrane was incubated with a secondary antibody (1:8000) at room temperature for 2 h. Protein bands were visualized by chemiluminescence and captured using a Tanon fully automatic chemiluminescence image analysis system (Model 5200 Multi, Tanon) with V2.0 software. The target protein bands were analyzed using Gel Pro Analyzer software (version 4.0).

### 2.9. Statistical analysis

All data are expressed as the mean ± standard deviation. One-way analysis of variance (ANOVA) with least significance difference post hoc multiple range tests was used to analyze data differences in SPSS 22.0 software (SPSS Inc., Chicago, IL, U.S.A.). Differences were considered significant at *P* < 0.05.

## 3. Results

### 3.1. Effects of ADP on serum liver function indicators AST and ALT

**Figure 1.**
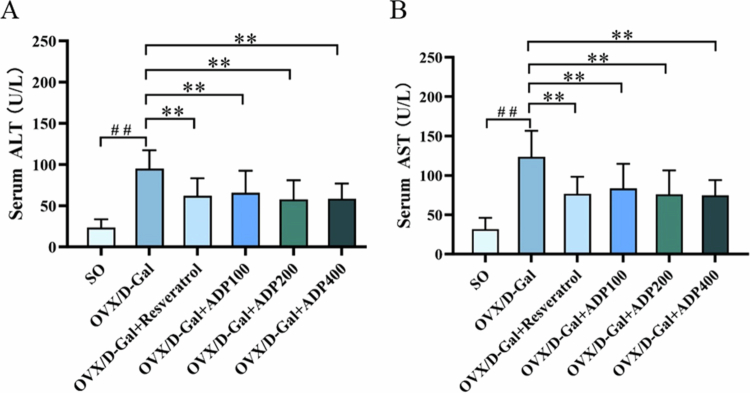
Effects of ADP on serum liver function indicators of AST and ALT activities. (A) The serum levels of ALT in different groups (mean ± SD, *n* = 10). (B) The serum levels of AST in different groups (mean ± SD, *n* = 10). Data were shown as mean ± SD (*n* = 10). Compared with the SO group, ^#^ *P* < 0.05, ^##^ *P* < 0.01; compared with the OVX/D-Gal group, *P < 0.05, **P < 0.01.

Compared with the SO group, the OVX/D-Gal group showed a significant increase in serum AST and ALT activity (*P* < 0.01), which is indicative of liver injury. However, resveratrol and ADP treatment for 49 days significantly decreased serum ALT and AST activity (*P* < 0.01) (Figure 1A–B), indicating that ADP regulated liver function to a certain extent and exerted hepatoprotective effects.

### 3.2. Effects of ADP on serum inflammatory factors TNF-*α*, IL-1β, and IL-6

**Figure 2.**
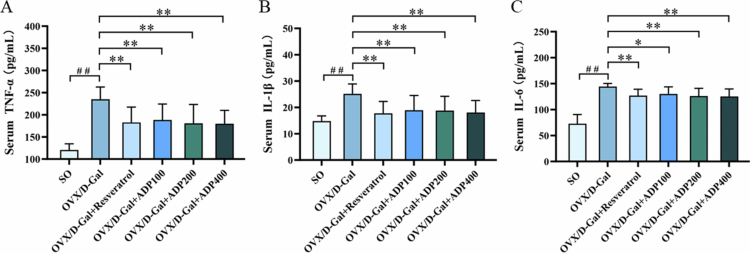
Effect of ADP on serum inflammatory factors of TNF-*α*, IL-1β, and IL-6 levels. (A-C) The serum level of TNF-*α*, IL-1β, and IL-6 in the rats (mean ± SD, *n* = 10). Data were shown as mean ± SD (*n* = 10). Compared with the SO group, ^#^ *P* < 0.05, ^##^ *P* < 0.01; compared with the OVX/D-Gal group, *P < 0.05, **P < 0.01.

Compared with the SO group, the OVX/D-Gal group exhibited significantly elevated serum TNF-*α*, IL-1β, and IL-6 (*P* < 0.01), indicating a systemic inflammatory response. However, resveratrol and ADP treatment significantly decreased these levels in comparison with those in the OVX/D-Gal group (*P* < 0.05) (Figure 2A–C), indicating that ADP effectively reduced proinflammatory cytokine levels and alleviated the OVX/D-Gal-induced inflammatory response.

### 3.3 Effects of ADP on oxidation and antioxidant biomarkers in the serum and liver tissue

**Figure 3.**
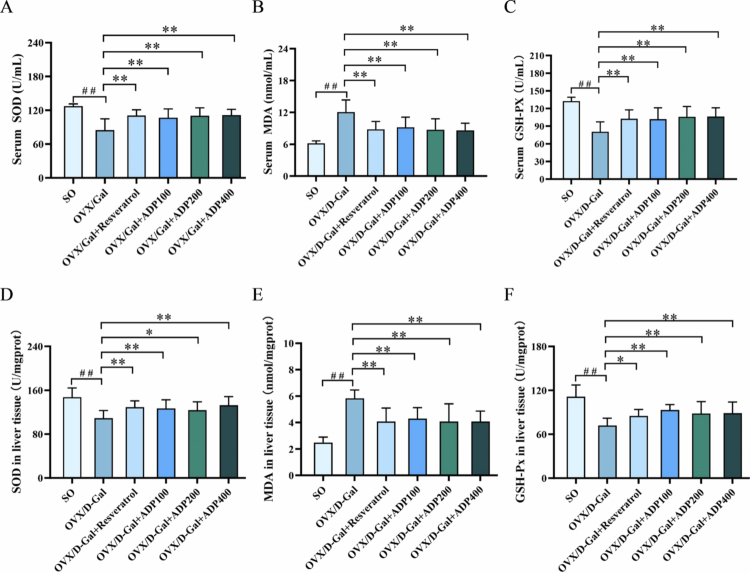
Effect of ADP on oxidation and antioxidant biomarkers in serum and liver tissue. (A–C) The serum level of SOD, MDA, and GSH-Px in the rats (mean ± SD, *n* = 10). (D–F) The liver tissue level of SOD, MDA, and GSH-Px in the rats (mean ± SD, *n* = 10). Data were shown as mean ± SD (*n* = 10). Compared with the SO group, ^#^ *P* < 0.05, ^##^ *P* < 0.01; compared with the OVX/D-Gal group, *P < 0.05, **P < 0.01.

Compared with the SO group, the OVX/D-Gal group exhibited a significant decrease in serum and liver antioxidant biomarker (SOD and GSH-Px) levels and a significant increase in serum and liver oxidative stress biomarker (MDA) levels (*P* < 0.01), indicative of oxidative stress. However, compared with the OVX/D-Gal group, the resveratrol and ADP groups exhibited significantly increased serum and liver tissue SOD and GSH-Px levels (*P* < 0.05), and significantly decreased MDA levels (*P* < 0.01) (Figure 3A–F). These findings indicate that ADP effectively attenuated oxidative stress and OVX/D-Gal-induced injury.

### 3.4. Effects of ADP on OVX/D-gal-induced hepatic histopathological changes

**Figure 4.**
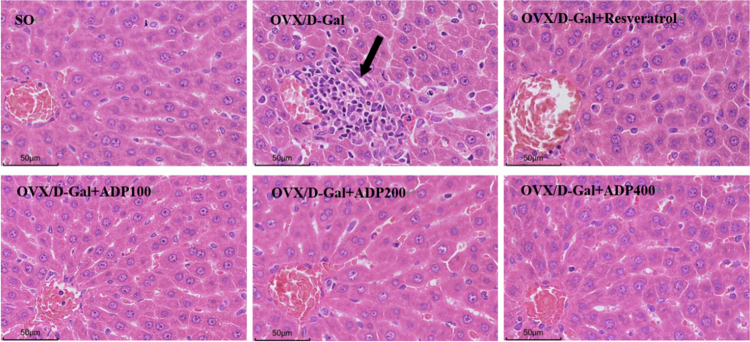
Effects of ADP on OVX/D-gal-induced hepatic histopathological changes (Hematoxylin-eosin staining, 400×). Note: the black arrow indicates focal necrosis of liver cells.

HE staining can visually demonstrate liver tissue damage, and the microscopic structure of liver tissue in the OVX/D-Gal group rats exhibited significant changes, including disorganized hepatocyte cords, focal necrosis, and inflammatory cell infiltration. Resveratrol and ADP treatment attenuated these changes: the hepatocyte cords demonstrated reduced hepatocyte death and were arranged nearly normally and in a radial mode; the level of focal necrosis was decreased; and the degree of inflammatory cell infiltration was alleviated (Figure 4). Thus, liver histopathology demonstrated improvement toward a normal appearance after ADP treatment.

### 3.5. Effects of ADP on the expression levels of oxidative stress-related genes in liver tissue

**Figure 5.**
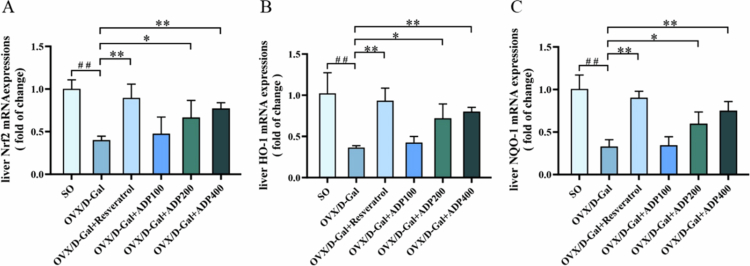
Effects of ADP on the expressions of oxidative stress-related genes in liver tissue. (A–C) The mRNA expressions of Nrf2, HO-1, and NQO-1 in rat liver tissue (mean ± SD, *n* = 3). Data were shown as mean ± SD (*n* = 3). Compared with the SO group, ^#^ *P* < 0.05, ^##^ *P* < 0.01; compared with the OVX/D-Gal group, *P < 0.05, **P < 0.01.

Compared with the SO group, the OVX/D-Gal group exhibited significantly downregulated Nrf2, HO-1, and NQO-1 mRNA expression in the liver (*P* < 0.01). In contrast, the resveratrol, ADP200, and ADP400 groups demonstrated significantly upregulated liver Nrf2, HO-1, and NQO-1 mRNA expression (*P* < 0.05) in comparison with that in the OVX/D-Gal group (Figure 5A−C). Thus, ADP treatment upregulated the mRNA expression of certain antioxidant genes.

### 3.6. Effects of ADP on the expression levels of oxidative stress-related proteins in liver tissue

**Figure 6.**
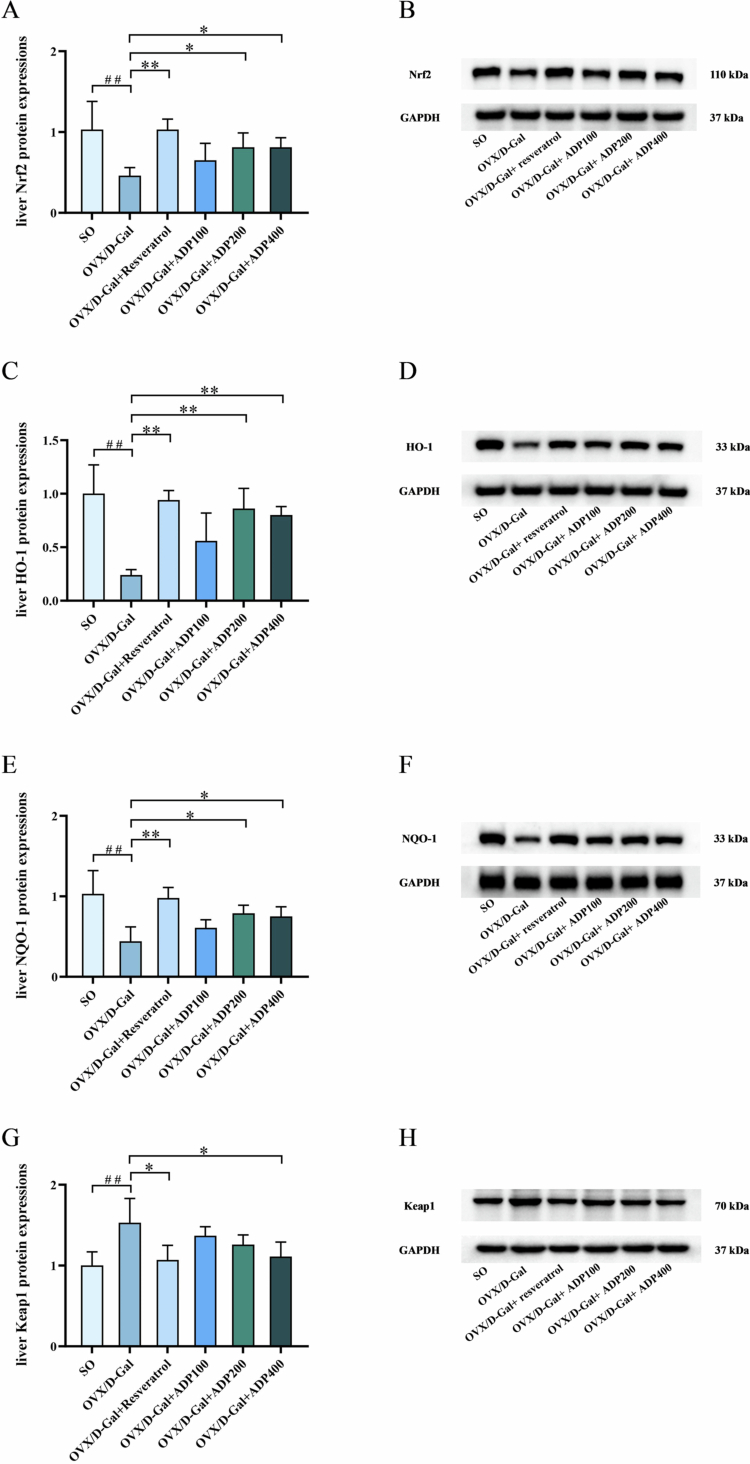
Effects of ADP on the expression of oxidative stress-related proteins in liver tissue. (A–H) The protein expression of Nrf2, HO-1, NQO-1, and Keap1 in rat liver tissue (mean ± SD, *n* = 3). Data were shown as mean ± SD (*n* = 3). Compared to SO group, ^#^ *P* < 0.05, ^##^ *P* < 0.01; compared to OVX/D-Gal group, *P < 0.05, **P < 0.01.

Compared with the SO group, the OVX/D-Gal group showed a significant upregulation of Keap1 protein expression in the liver, accompanied by a significant downregulation of Nrf2, HO-1, and NQO-1 protein expression (*P* < 0.01). Treatment with resveratrol and ADP400 significantly downregulated Keap1 expression (*P* < 0.05), compared with that in the OVX/D-Gal group, whereas resveratrol, ADP100, and ADP400 significantly upregulated Nrf2, HO-1, and NQO-1 expression (*P* < 0.05) (Figure 6A–H). These findings suggest that ADP attenuated OVX/D-Gal-induced oxidative stress in rats, at least in part, by enhancing the activation of the Nrf2/HO-1/NQO-1 signaling pathway.

### 3.7. Effects of ADP on the expression levels of apoptosis-related proteins in liver tissue

**Figure 7.**
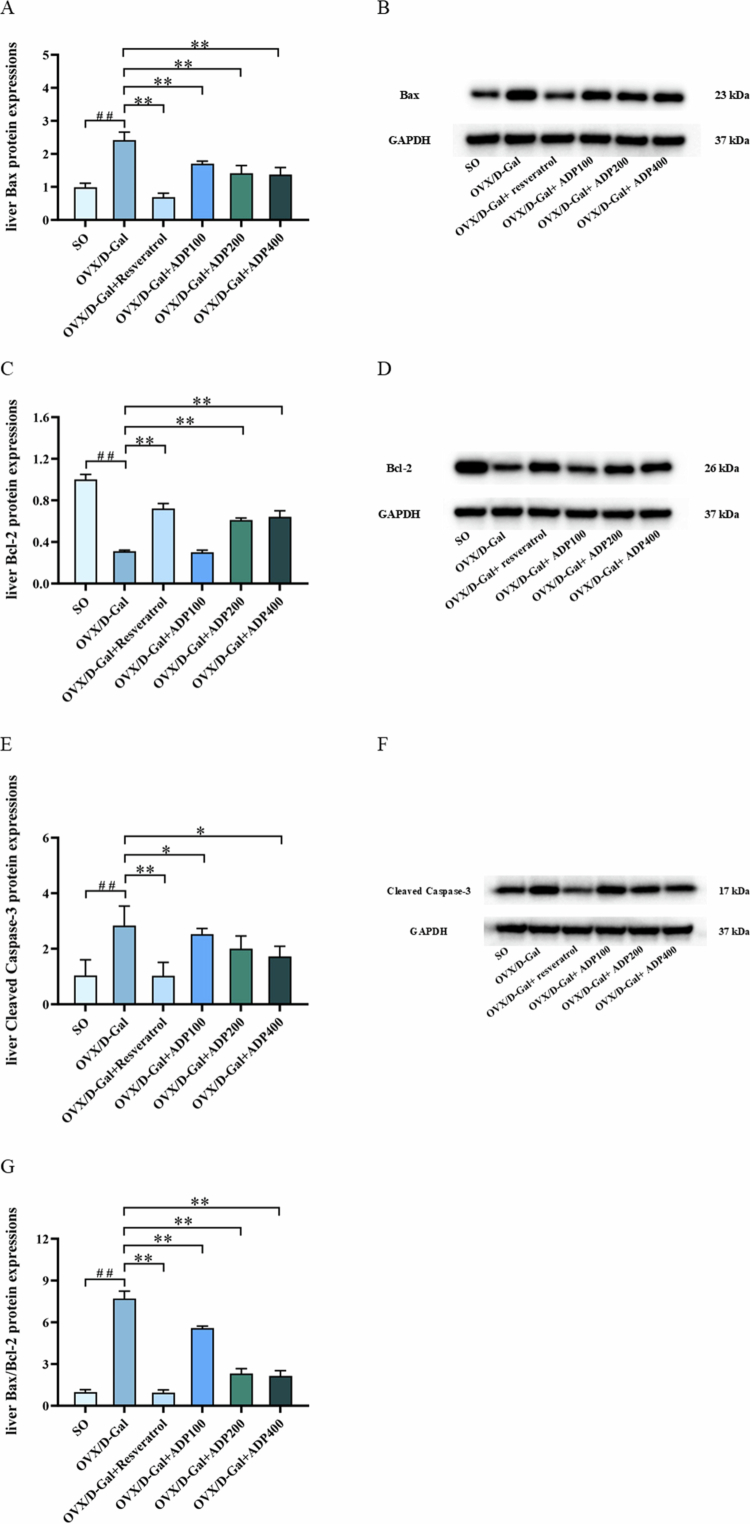
Effects of ADP on the expressions of apoptosis-related proteins in liver tissue. (A–G) The protein expressions of Bax, Bcl-2, Bax/Bcl-2, and Cleaved caspase-3 in rat liver tissue (mean ± SD, *n* = 3). Data were shown as mean  ± SD (*n* = 3). Compared with the SO group, ^#^ *P* < 0.05, ^##^ *P* < 0.01; compared with the OVX/D-Gal group, *P < 0.05, **P < 0.01.

Compared with the SO group, the OVX/D-Gal group exhibited significantly downregulated Bcl-2 expression in the liver (*P* < 0.01), along with significantly elevated Bax, Bax/Bcl-2 ratio, and cleaved caspase-3 levels (*P* < 0.01). The resveratrol, ADP200, and ADP400 groups demonstrated significantly higher liver Bcl-2 expression (*P* < 0.05) than that in the OVX/D-Gal group. Significantly reduced Bax expression levels and Bax/Bcl-2 ratios (both *P* < 0.01) were observed in the resveratrol and ADP groups, and liver cleaved caspase-3 levels were significantly downregulated (*P* < 0.05) in the resveratrol, ADP100, and ADP400 groups (Figure 7A–G). Taken together, these findings indicate that ADP treatment attenuated OVX/D-Gal-induced apoptosis in the liver by regulating the expression of apoptosis-related proteins.

## 4. Discussion

In this study, an OVX/D-Gal-induced liver injury animal model was successfully established. It is well established that ALT and AST are key indicators of hepatocyte damage, and the levels of these indicators can reflect whether liver injury has occurred [37,38]. Mild liver damage can lead to increased serum ALT levels, whereas severe damage can cause liver cell necrosis, impairing mitochondrial function and leading to the release of AST [39]. In this study, rats in the OVX/D-Gal group exhibited significantly elevated serum ALT and AST levels and severe pathological hepatic changes, including disorganized hepatic cord architecture, focal necrosis, and inflammatory cell infiltration. ADP demonstrated efficacy similar to that of the positive control, resveratrol, in reducing ALT and AST levels and improving liver pathological scores, with no significant differences between the two drugs, indicating that ADP alleviate OVX/D-Gal-induced liver injury and possesses hepatoprotective potential comparable to resveratrol.

The liver, as the central organ for metabolism and detoxification, is a primary target of oxidative free radical attack, which triggers oxidative stress [14,15,40]. Oxidative stress plays a core role in liver injury, and antioxidant intervention is a key strategy for protecting hepatocytes and delaying liver disease progression [41,42]. Natural products, which are characterized by multi-component and multi-target synergistic effects, high safety, and few side effects, are considered good alternatives to synthetic chemical drugs for treating liver injury [43,44]. SOD, GSH-Px, and MDA are major antioxidant-related substances and serve as key indicators in oxidative and antioxidant research [45]. SOD and GSH-Px are highly efficient antioxidant enzymes that scavenge oxygen free radicals and effectively reduce intracellular lipid peroxidation reactions; thus, their activity levels can indirectly reflect a rat’s ability to eliminate free radicals. MDA is an end product of lipid peroxidation, and its level reflects the degree of free radical-induced damage [46–48]. In this study, OVX/D-Gal rats exhibited decreased liver SOD and GSH-Px levels and increased MDA levels, indicating reduced liver antioxidant capacity and exacerbated lipid peroxidation. ADP intervention reversed the above indicators, confirming its antioxidant activity. This finding is consistent with the protective effects of natural polysaccharides, such as *Polygonatum sibiricum* [26] and *Acanthopanax senticosus* polysaccharides [49], in D-Gal models, further indicating that polysaccharide compounds can resist oxidative damage by enhancing endogenous antioxidant capacities.

Oxidative stress and inflammatory responses are closely intertwined, acting as mutual catalysts that collectively drive the progression of liver injury [50]. In this study, the systemic inflammatory status was further evaluated. The serum TNF-*α*, IL-1β, and IL-6 levels were significantly elevated in the OVX/D-Gal group compared with those in the SO group, indicating a systemic inflammatory response. This effect was significantly alleviated by ADP treatment, demonstrating that ADP effectively reduces proinflammatory cytokine levels and alleviate OVX/D-Gal-induced inflammatory responses. Taken together with the aforementioned results showing that ADP reduces ALT and AST levels, ameliorates liver histopathology, and enhances antioxidant capacity, it is evident that ADP exerts a synergistic hepatoprotective effect by simultaneously inhibiting oxidative stress and inflammation.

The Nrf2/HO-1/NQO-1 pathway is a classic antioxidant signaling pathway involved in antioxidant stress responses closely related to maintaining the intracellular redox balance and protecting against oxidative stress damage [51]. Nrf2 is a key transcription factor that regulates the oxidative stress response, and its upregulation enhances the cellular antioxidant capacity. Under oxidative stress, the upstream inhibitory molecule Keap1 undergoes conformational changes or expression downregulation. Promoting its dissociation from Nrf2 leads to its nuclear translocation, which induces the expression of downstream antioxidant enzymes, such as HO-1 and NQO-1, thereby enhancing the cellular antioxidant defense system [52,53]. Numerous studies have demonstrated that various compounds can alleviate oxidative stress and exert hepatoprotective effects through the Nrf2/HO-1/NQO-1 pathway. For example, curcumin has been shown to attenuate oxaliplatin-induced liver injury and oxidative stress by activating the Nrf2 pathway [54]. Similarly, specnuezhenide alleviates CCl4-induced liver injury in mice by inhibiting oxidative stress and Nrf2 signaling [55]. In the present study, ADP was found to upregulate liver Nrf2, HO-1, and NQO-1 mRNA and protein expression, while downregulating liver Keap1 protein expression, in OVX/D-Gal-induced liver injury model rats. Resveratrol is a classic activator of the Nrf2 pathway, and its hepatoprotective effect mainly depends on transcriptional regulation rather than direct ROS scavenging [56]. This study demonstrated that ADP exhibits efficacy comparable to that of resveratrol, suggesting that its mechanism may involve the upregulation of the endogenous antioxidant enzyme system. Notably, OVX-induced estrogen deficiency may downregulate the Nrf2 signaling pathway, thereby increasing the liver’s susceptibility to oxidative stress [57,58]. The hepatoprotective effect of ADP may be partly achieved by ‘substituting’ for estrogen function and restoring Nrf2 pathway transcriptional activity. ADP may upregulate the endogenous antioxidant enzyme system primarily through transcriptional regulation, rather than direct chemical scavenging. However, whether ADP, as a polysaccharide with a large molecular weight, directly binds to Nrf2 or its inhibitor Keap1, remains unclear. Guo [59], Wang [60], and other researchers have revealed how natural products interact with key proteins in the oxidative signaling network using molecular docking techniques, providing an important methodological reference for subsequent investigations into the direct molecular targets of ADP.

Apoptosis plays a significant role in maintaining tissue homeostasis and development [61,62]. Oxidative stress directly damages cells and plays a key role in inducing apoptosis [63]. The Bcl-2 family is a crucial regulator of apoptosis, among which Bax is pro-apoptotic, and Bcl-2 is anti-apoptotic. The balance between the expression levels of these two proteins determines cell survival or death upon apoptotic stimulation, and their ratio is a key indicator of apoptosis [64,65]. In this study, the expression of anti-apoptotic Bcl-2 was downregulated in the liver tissue of OVX/D-Gal rats, whereas that of pro-apoptotic Bax and cleaved caspase-3 was increased, as was the Bax/Bcl-2 ratio. ADP intervention reversed this trend. These results indicate that inhibiting hepatocyte apoptosis is another important mechanism by which ADP alleviates liver injury. However, programmed cell death includes pathways other than apoptosis, such as pyroptosis. Pyroptosis, as a caspase-mediated inflammatory cell death, has been implicated in oxidative liver injury [66]. The NOD-like receptor thermal protein domain-associated protein 3 (NLRP3) inflammasome is a key regulatory complex in pyroptosis. NLRP3 activation leads to the cleavage and activation of caspase-1, which subsequently cleaves gasdermin D and releases proinflammatory cytokines, ultimately inducing pyroptosis [67]. Notably, Wang et al. [68] demonstrated that, in an atherosclerosis model, the natural compound isoorientin reduces the level of lysine demethylase 4A, thereby restoring SCF E3 ubiquitin ligase complex-mediated NLRP3 ubiquitination, which inhibits macrophage pyroptosis and alleviates lesion progression. The aforementioned study revealed a novel mechanism of NLRP3 ubiquitination regulation in the pyroptosis pathway and provided evidence for the protective effects of natural products by targeting pyroptosis. Recent studies have indicated that NLRP3 inflammasome-mediated pyroptosis plays a critical role in oxidative liver injury [69,70]. Furthermore, in the present study, ADP was shown to reduce the levels of TNF-*α*, IL-1β, and IL-6. The secretion of mature IL-1β depends on NLRP3 inflammasome activation [71]. Thus, ADP might, at least in part, alleviate OVX/D-Gal-induced liver injury by interfering with NLRP3 inflammasome activation and the downstream pyroptosis pathway. However, this hypothesis remains to be rigorously validated in future investigations.

A complex regulatory network exists between oxidative stress and autophagy. Autophagy can clear damaged mitochondria and excess ROS, thereby protecting against oxidative stress [72,73]. Chen et al. [74] showed that davunetide enhances autophagy and inhibits necroptosis via SIRT1-FOXO1-TFEB in a neuroprotection model, suggesting that a similar mechanism may operate in liver injury. Whether ADP induces autophagy requires further study. The restoration of redox homeostasis involves crosstalk with cellular metabolism. Recent studies have revealed the critical role of ‘redox-metabolism crosstalk’ in cytoprotection. Zhang et al. [75] found that M2 macrophage-derived exosomes mediate metabolic reprogramming of macrophages (inhibiting glycolysis and maintaining oxidative phosphorylation) by regulating the PKM2/HIF-1α axis, and promoting M2 polarization, thereby exerting a protective effect in viral myocarditis. Pang et al. [76] showed that Lawsone could enhance the activity of the pentose phosphate pathway and remodel the glucose metabolism network of T cells through NQO1-catalyzed redox cycling, endowing T cells with long-term antitumor capability. Thus, whether ADP also synergistically enhances the antioxidant defense capacity by regulating hepatocyte metabolic reprogramming warrants further investigation. Finally, regarding systemic biomarkers, Hui et al. [77] indicated that monitoring plasma protein markers could help assess treatment responses and disease progression. Thus, subsequent studies could employ proteomics techniques to identify specific changes in plasma proteins after ADP treatment, thereby providing more comprehensive pharmacodynamic evaluation indicators for clinical application.

In conclusion, ADP attenuates OVX/D-Gal-induced liver injury. Nevertheless, its therapeutic potential in chronic liver disease models, such as fibrosis and cirrhosis, remains to be determined and merits further study. Furthermore, the hypothesized involvement of pyroptosis, autophagy, metabolic crosstalk, and systemic biomarkers is currently speculative and necessitates confirmation through targeted experimental investigations.

## 5. Conclusion

In summary, this study demonstrates that ADP activates the Nrf2/HO-1/NQO-1 signaling pathway, enhances hepatic antioxidant capacity, inhibits oxidative stress-mediated apoptosis, and attenuates systemic inflammation, thereby alleviating OVX/D-Gal-induced liver injury. These findings highlight ADP as a promising natural polysaccharide-based hepatoprotective agent with multi-target synergistic effects and a favorable safety profile. However, the direct molecular target of ADP in liver injury remains unclear, and its protective effects against chronic liver diseases and extrahepatic organ injuries remain to be validated. Future studies should employ molecular docking and proteomic approaches to identify the direct binding targets of ADP, evaluate its therapeutic potential in chronic liver injury models, and further explore its possible roles in regulating pyroptosis, autophagy, and metabolic reprogramming.

## Supplementary Material

Supplementary Material ARRIVE guidelines.docx

## Data Availability

All relevant study data are included in the article and available from the corresponding author on reasonable request.
